# Enhanced mm-Wave Frequency Up-Conversion via a Time-Varying Graphene Aperture on a Cavity Resonator

**DOI:** 10.3390/mi16060679

**Published:** 2025-06-04

**Authors:** Stamatios Amanatiadis, Theodosios Karamanos, Fabrice Lemoult, Nikolaos V. Kantartzis

**Affiliations:** 1 School of Electrical and Computer Engineering, Aristotle University of Thessaloniki, 54124 Thessaloniki, Greece; kant@auth.gr; 2 Laboratoire GeePs, Sorbonne Université, CNRS, 75005 Paris, France; theodosios.karamanos@sorbonne-universite.fr; 3 Laboratoire GeePs, Université Paris-Saclay, CentraleSupélec, CNRS, 91190 Gif-sur-Yvette, France; 4 Institut Langevin, ESPCI Paris, Université PSL, CNRS, 75006 Paris, France; fabrice.lemoult@espci.psl.eu

**Keywords:** graphene antenna, mm-wave, non-linear antenna, substrate-integrated cavity, time-varying surfaces

## Abstract

The transition to 5G and beyond has highlighted the need for efficient devices that operate at mm-wave frequencies, which require new structures and pose fabrication challenges. This paper proposes a novel non-linear antenna that combines the well-established substrate-integrated cavity (SIC) radiators and time-varying graphene for generating harmonic frequencies in the mm-wave spectrum. Graphene is represented as having a dispersive surface conductivity, while time modulation of the conductivity is introduced by varying the applied bias electric field. A modified FDTD algorithm is, additionally, used to simulate the time-varying graphene behaviour under different modulation schemes. The final antenna design involves an SIC resonator with a graphene-covered slot aperture for radiation. The numerical study highlights the effective generation of harmonics using the modulated graphene at the mm-wave regime. Finally, different modulation schemes are applied to enhance certain higher-order harmonics, demonstrating the potential of this non-linear antenna design for future mm-wave and THz frequency applications.

## 1. Introduction

During the past years, the transition to 5G as well as ongoing research towards telecommunication systems beyond current standards have indicated a pressing need for new and efficient devices. In particular, the transition from radio and microwave to mm-wave frequencies requires devices that operate at higher frequencies to provide larger bandwidths. Consequently, mm-wave devices possess finer structures that pose challenges to the fabrication processes. Thus, to facilitate the fabrication of novel waveguiding designs in the mm-wave spectrum, the substrate-integrated waveguide (SIW) structure has been proposed [1,2]. An SIW consists of two parallel dense series of metallic vias into metallic-covered substrate, which, in turn, enables wave propagation in the channel created by the vias–metallic cover system. This concept has been further utilised in various designs, like frequency filters [3], couplers [4], or power dividers [5]. Furthermore, the SIW concept was expanded for antenna applications through the substrate-integrated cavity (SIC) design [6,7]. An SIC works as a cavity resonator antenna composed of a closed SIW design, fed with a coaxial cable at the bottom and with an aperture on top [8,9,10,11]. Many interesting features arise with this design at the mm-wave frequencies, including easier fabrication on dielectric substrates, while enabling an easier attachment of advanced materials on the radiative slot.

Another concept that has recently gained the attention of researchers and engineers is that of time-varying materials [12,13,14,15,16]. Time-varying materials are artificial media characterised by rapid changes in their electromagnetic properties while a slower-paced phenomenon takes place. Time-varying artificial media or surfaces hold promise for the engineering of various novel and advanced devices, outperforming common ones that are stationary in time for different spectra, from microwaves to optics. Applications include, among others, parametric amplifiers [17,18,19], frequency shifters [20,21], anti-reflective surfaces [22], and reconfigurable, beam-forming metasurfaces [23,24]. A particular feature of periodically modulated time-varying media is the incitement of harmonic frequencies, which could be very interesting for designing new sources for the next generation of telecommunications in the mm-wave spectrum. In this context, graphene is a perfect candidate for the time-varying platform in the mm-wave and the THz spectrum, because its surface conductivity can be effectively and rapidly modulated using an externally applied electric bias field. Due to its impressive characteristics, this 2D material has been theoretically analysed and utilised in its time-varying form for frequency harmonic generation beyond the far-infrared regime [25,26,27,28]. Consequently, the exploitation of the inherent time-varying capabilities of graphene can be combined with a conventional radiator, such as the cavity antenna, to produce a frequency generator device in a straightforward manner.

In this paper, we combine the concepts of the SIC radiator and the time-varying graphene to propose a non-linear antenna design, capable of generating and radiating frequencies in the mm-wave spectrum. In particular, the inherent ability of graphene modulation via an external bias is integrated with a well-established antenna, providing a realistic source for modern telecommunications. Initially, we describe graphene with a dispersive surface conductivity following the Drude model. Afterwards, we introduce the time modulation of the surface conductivity by alternating the real-time bias electric field imposed on graphene. Then, we utilise an efficient and accurate modified finite-difference time-domain (FDTD) algorithm to numerically simulate the time-varying surface conductivity via the concept of the equivalent surface current. Initially, the theoretical case of an infinite time-varying graphene sheet is simulated to examine the scattering effects for a harmonic periodic modulation and gain insight into the planar material’s behaviour. Following the theoretical analysis, we optimally design an SIC to operate approximately at 60 GHz, while we draw a suitable graphene-loaded slot aperture on its upper side to enable radiation. The developed numerical algorithm is now employed to investigate the time-varying aspects of the proposed non-linear antenna. Numerical results prove the reasonable higher-order harmonic generation, while different types of periodic modulation schemes are applied to enhance a certain set of desired harmonics. As a consequence, this analysis certifies the potential of our design for future non-linear antennas and frequency generators for the mm-wave and THz regimes.

## 2. Theoretical Formulation

### 2.1. Graphene’s Surface Conductivity

In our work, graphene is considered as a thin layer, characterised by its surface conductivity $\sigma_{\mathrm{gr}}$. The latter depends on the chemical potential $\mu_{c}$, controlled by an external bias electric field $E_{\mathrm{bias}}$, the scattering rate Γ, related to the main loss mechanism, and the temperature *T*. The surface conductivity is evaluated by the compact expression resulting from the Kubo formula [29], involving only the intraband term, which is the dominant term in the mm-wave spectrum, as follows:

(1) $$\sigma_{\mathrm{gr},\mathrm{intra}}=\frac{A_{\mu_{c}}}{j\omega +2\Gamma },\;A_{\mu_{c}}=\frac{q_{e}^{2}\;k_{B}T}{\pi \hbar^{2}}\left[\frac{\mu_{c}}{k_{B}T}+2\ln\left(1+e^{\;-\mu_{c}/k_{B}T}\right)\right],$$

with $A_{\mu_{c}}$ as the frequency-independent term, $q_{e}$ as the electron charge, and *ħ*, $k_{B}$ the reduced Planck and Boltzmann constants, respectively. We can observe in (1) that the surface conductivity of graphene follows the Drude dispersion model. As mentioned above, the chemical potential is connected with the applied electrostatic bias field, $E_{\mathrm{bias}}$, by the following:

(2) $$E_{\mathrm{bias}}=\frac{q_{e}}{\varepsilon_{0}\pi \hbar^{2}u_{F}^{2}}\;\int_{0}^{\infty }x\left[f_{d}\left(x\right)-f_{d}\left(x+2\mu_{c}\right)\right]\;dx,$$

where $u_{F}$ is the Fermi velocity, while $f_{d}\left(x\right)$ follows the Fermi–Dirac distribution, or

(3) $$f_{d}\left(x\right)=\frac{1}{1+e^{\;\left(x-\mu_{c}\right)/k_{B}T}}\;.$$

**Figure 1:**
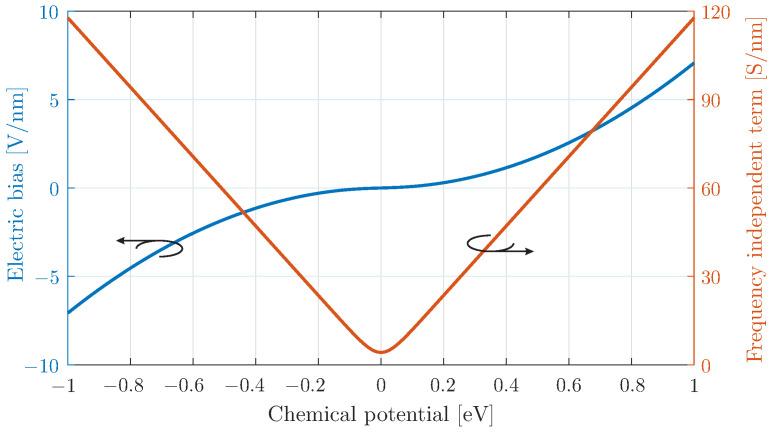
Graphene chemical potential as a function of the electric bias field and the frequency-independent term of surface conductivity via (2) and (1), respectively.

The relation between the chemical potential with its bias field is depicted in Figure 1, indicating a strongly non-linear response near bias field values close to zero. This is more evident in the relation of the chemical potential with the frequency-independent term $A_{\mu_{c}}$ of the surface conductivity. Here, a linear approximation is valid for $|\mu_{c}|$ > 0.1 eV. In practice, the modulation scheme can be applied on the external bias field $E_{\mathrm{bias}}$. This will result in a dynamic alteration of the chemical potential $\mu_{c}$, evaluated through (2) and the frequency-independent term $A_{\mu_{c}}$ of the surface conductivity in (1). This procedure is able to realistically account for the non-linearities of chemical potential near the zero values of the applied bias field. Note that the graphene surface conductivity in (1) has a low-pass filter response, which can potentially degrade the stimulation of higher frequencies. Nevertheless, the cut-off frequency, defined by the scattering rate Γ, is beyond 10 THz, while our analysis is focused at the much lower mm-wave regime.

In this context, the temporal modulation of graphene is realistically applied as

(4) $$E_{\mathrm{bias}}\left(t\right)=E_{0}+E_{m}\left(t\right),$$

where $E_{0}$ is a constant bias, and $E_{m}\left(t\right)$ is the time-varying component of the bias. In this paper, we investigate the following three distinct cases of periodic temporal variations, with $\omega_{m}$ denoting the modulation frequency:

1. $E_{\mathrm{bias}}\left(t\right)=E_{0}+m_{0}\;E_{0}\cos\left(\omega_{m}t\right)$, namely a harmonic variation, where $m_{0}$ is the modulation depth, characterising the strength of the modulation;
2. $E_{\mathrm{bias}}\left(t\right)=E_{0}+m_{0}\;E_{0}\;\mathrm{sgn}\left\{\cos\left(\omega_{m}t\right)\right\}$, where sgn{·} is the sign function, representing a square wave or periodic pulse modulation;
3. $E_{\mathrm{bias}}\left(t\right)=E_{0}\;\cos\left(\omega_{m}t\right)$, representing a harmonic modulation without a constant bias, namely around the minimum surface conductivity.

The first type of time modulations is the same that was used in previous modelling attempts for time-varying and dispersive media [17,26,30,31]. Similarly, the numerator of the dispersion equation, Drude [26] or Lorentz [31], was harmonically modulated in the same way as the first type above. In this work, we further expand this analysis scheme by examining more modulation types in dispersive media and by applying the findings on non-linear graphene antenna structures.

### 2.2. FDTD Modelling of Graphene as a Surface Conductivity

The analysis throughout this work is conducted numerically via an accurate FDTD method, since its time-domain nature is perfectly suited for simulation of time-varying media. The FDTD modelling of the two-dimensional material is realised via the equivalent surface current ${\vec{J}}_{\mathrm{gr}}=\sigma_{\mathrm{gr},\mathrm{intra}}\vec{E}$. In particular, for a graphene layer on the xy-plane, the surface current is calculated at the time domain as

(5) $${\vec{J}}_{\mathrm{gr}}=\frac{A_{\mu_{c}}}{j\omega +\Gamma }\;\vec{E}\Rightarrow \frac{\partial {\vec{J}}_{\mathrm{gr}}}{\partial t}+\Gamma \;{\vec{J}}_{\mathrm{gr}}=A_{\mu_{c}}\vec{E}\;.$$

Afterwards, an Auxiliary Differential Equation (ADE) scheme [32] is applied to generate the surface current updating equation, e.g., for the *x* component:

(6) $${\left.J_{x}\right|}^{n+\frac{1}{2}}=\frac{1-\Gamma \Delta t}{1+\Gamma \Delta t}{\left.J_{x}\right|}^{n-\frac{1}{2}}+\frac{{\left.A_{\mu_{c}}\right|}^{n}}{1+\Gamma \Delta t}{\left.E_{x}\right|}^{n},$$

where the surface current component lies in an identical position as the one with the corresponding electric field at the Yee cell, and Δt is the FDTD time-step. Then, the contribution of the graphene surface current is introduced in the FDTD, updating the equations through a finite-difference approximation of the Ampère law as follows:

(7) $$E_{x}{|}^{n+1}=E_{x}{|}^{n}+\frac{\Delta t}{\varepsilon }\left(\frac{\partial }{\partial y}H_{z}{|}^{n+\frac{1}{2}}-\frac{\partial }{\partial z}H_{y}{|}^{n+\frac{1}{2}}\right)-\frac{\Delta t}{\varepsilon \Delta y}J_{x},\mathrm{gr}{|}^{n+\frac{1}{2}},$$

where Δz is the cell size across *z* and normalises the graphene surface current to enable its two-dimensional nature. A similar expression is applied for the *y* surface current component, while the rest of the updating equations are kept in the traditional manner. Note that the explicit nature of the FDTD approach combined with the ADE scheme is more efficient in comparison to other Finite Element Time-Dependent methods employed in related work [17,31], where considerable computational power and time are required to provide numerical simulation results.

Finally, the time-varying features are calculated in the abovementioned procedure via a variation of the frequency-independent term $A_{\mu_{c}}{|}^{n}$ in (6) as follows:

(8) $${\left.A_{\mu_{c}}\right|}^{n}=\frac{q_{e}^{2}\;k_{B}T}{\pi \hbar^{2}}\left[\frac{\mu_{c}{|}^{n}}{k_{B}T}+2\ln\left(1+e^{-\mu_{c}{|}^{n}/k_{B}T}\right)\right],\;\mu_{c}{|}^{n}=E_{\mathrm{bias}}\left(n\Delta t\right).$$

A brief outline of the procedure is as follows:

- At *n*: update of the electric components and the frequency-independent term.
- At $n+\frac{1}{2}$: update of the magnetic and the surface current components.

### 2.3. Frequency Generation Using a Time-Varying Graphene Sheet

Initially, the simple case of a plane wave normally incident on a time-varying graphene sheet was studied. This scenario was chosen to evaluate the frequency up-conversion properties without the influence of complex geometries. To this end, a robust one-dimensional FDTD algorithm was employed, with a central frequency of $f_{0}=60\;\mathrm{GHz}$ selected to align with the n263 5G band [33]. The graphene scattering rate was fixed at Γ=0.33meV, and two different constant electric biases were considered: $E_{0,1}=85\;V/\mu m$ and $E_{0,2}=300\;V/\mu m$, corresponding to chemical potentials $\mu_{c,1}=0.1\;\mathrm{eV}$ and $\mu_{c,2}=0.2\;\mathrm{eV}$, respectively. Finally, harmonic variation of the electric bias was utilised, with the modulation depth set to $m_{0}=0.9$, while the modulation frequency was $\omega_{m}=2\pi \times 10$ GHz, or $\omega_{m}=\omega_{0}/6$.

**Figure 2:**
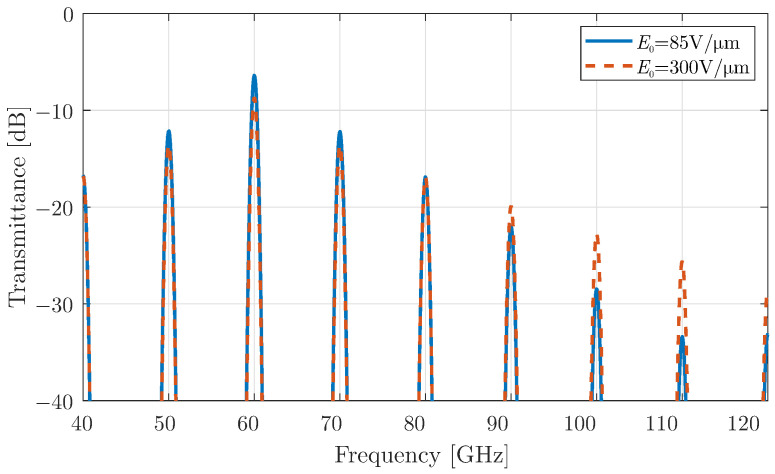
Transmission coefficient of a plane wave that propagates normally towards a time-varying graphene layer with modulation strength $m_{0}=0.9$.

The results of the transmitted or scattered wave for the time-modulated surface are shown in Figure 2, highlighting the generation of harmonics lower and higher than the central one. In particular, the generated harmonics are observed at $\omega_{n}=\omega_{0}\pm n\omega_{m}$, as reported in previous studies of time-varying media [12,14,15]. Although the transmitted power decreases with increasing harmonic order, this effect is more pronounced for the smaller bias value. This behaviour is attributed to the near-zero conductivity differentiation at lower chemical potentials, as explained in Figure 1. Therefore, the effect of higher-order harmonic scattering from time-varying surfaces, including a modulated graphene sheet, can be further utilised in more complex design concepts, such as non-linear antennas.

## 3. Cavity Resonator with Graphene-Coated Aperture

**Figure 3:**
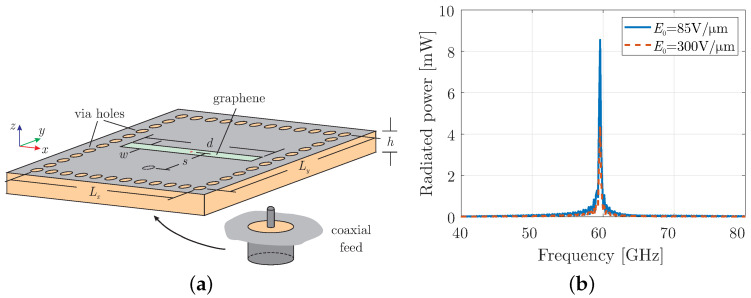
(**a**) The geometry of the substrate-integrated cavity with graphene-covered opening and (**b**) total radiated power using only a constant bias field.

After demonstrating the properties of time-varying graphene in the previous section, we now create a non-linear antenna by placing a time-modulated graphene on a cavity resonator structure. The cavity resonator is, herein, implemented using SIW technology [1,7] as illustrated in Figure 3a. The structure is composed of two perfect electric conductor (PEC) layers on the two sides of the RT/duroid 5880 substrate, with a thickness of h=0.127 mm, a relative permittivity of $\varepsilon_{r}=2.2$, and a dissipation factor of tanδ=0.0009. Metallic-coated via holes are drilled inside the substrate, with a diameter of $d_{h}$ = 100 μm and a pitch of $t_{h}$ = 210 μm, forming a cavity with dimensions $L_{x}=L_{y}=1.7$ mm. The excitation for the cavity is realised by inserting the core of a coaxial connector in the substrate located at distance $s=L_{y}/2=1.2$ mm from the centre towards the y-axis. Finally, an aperture is formed at the top layer with dimensions of d=1 mm and w = 90 μm, which is covered by graphene with scattering rate set to Γ=0.33 meV at room temperature. Note that the proposed structure can be designed in a conformal manner since the confinement functionality of the via holes is not degraded by the curvature [34,35], while graphene can, also, operate conformally [36]. Finally, it must be mentioned that the analysis is purely numerical since graphene is beyond the fabrication capabilities of our laboratory.

The FDTD simulation domain is discretised into 165×165×125 mesh cells with a cell size of Δx=Δy=Δz = 27 μm and a time-step of 50 fs. Open boundaries are truncated using a 16-cell thick Perfectly Matched Layer (PML), and the stimulation is achieved with a broadband pulse centred at 60 GHz and an input power of 100 mW.

Initially, the cavity with a graphene layer at its aperture is simulated without time modulation to evaluate its conventional performance. The total radiated power is shown in Figure 3b, confirming the cavity antenna’s operation at the desired frequency of $f_{0}$ = 60 GHz. Notably, the radiated power is lower than the input power due to the conductive properties of graphene in the millimeter-wave regime. This reduction becomes more pronounced under a bias field of $E_{0}$ = 300 V/μm, as the surface conductivity of graphene increases with the absolute value of the chemical potential. In the next subsections, the three modulation types, introduced in the previous section, are implemented in the proposed cavity resonator, and their effects are discussed using a modulation frequency $\omega_{m}=\omega_{0}$/6 = 2*π* × 10 GHz.

### 3.1. Harmonic Modulation of Graphene Using Constant Bias

**Figure 4:**
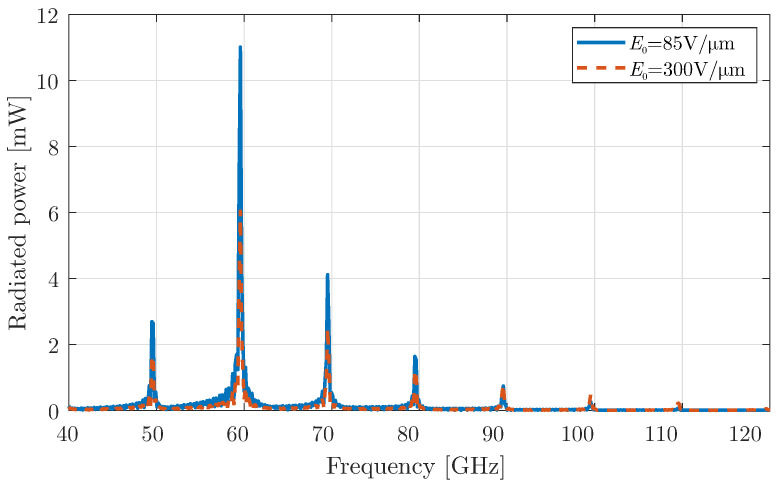
Total radiated power for a graphene layer with harmonic modulation using a constant bias field and modulation strength $m_{0}=0.9$.

At this stage, the first type of harmonic modulation, discussed in Section 2.1, is activated in the graphene-coated aperture, and the corresponding simulation results are presented in Figure 4. This modulation mechanism allows the antenna to shift its operating frequencies beyond the fundamental mode. Specifically, the up-converted frequencies correspond to higher harmonic components, which become visible as a result of the time modulation of the graphene aperture.

It is important to note that when the bias field is set to a lower constant value, a larger portion of the radiated power is concentrated at the fundamental frequency and the low-order harmonics. However, as predicted in Section 2.3, this occurs at the expense of power at the higher harmonic frequencies, where the radiated power decreases significantly. This trend aligns with the theoretical predictions and validates the system’s behaviour under varying bias conditions. To better understand this trade-off in power distribution across harmonics, a detailed investigation is conducted by analysing the modulation depth at different bias field values.

**Figure 5:**
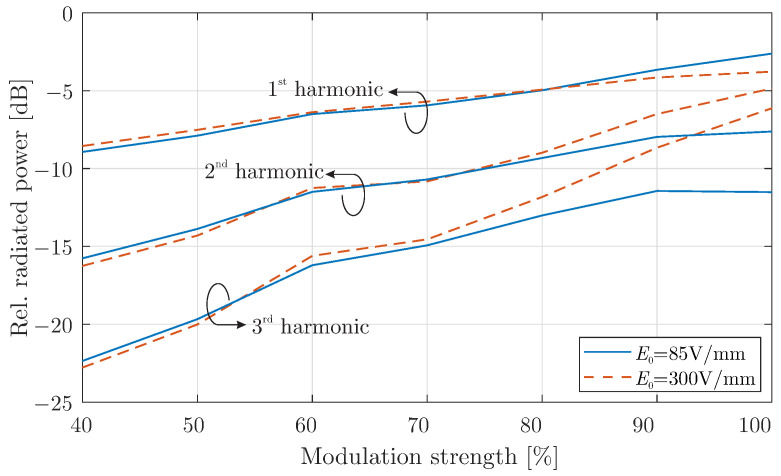
Radiated power of harmonics compared to the one at the fundamental frequency for different modulation strength values.

In Figure 5, the relative radiated power, defined as the ratio of the harmonic power to the power at the fundamental frequency, is plotted as a function of the modulation depth. The results indicate that when the modulation strength exceeds 90% or $m_{0}=0.9$, a noticeable shift in the relative power distribution across harmonic components occurs. Specifically, beyond this modulation threshold, the power associated with higher harmonics begins to increase significantly. As the modulation depth is further amplified, the radiated power at higher harmonics continues to grow, enhancing the up-conversion process. This demonstrates that modulation depth is a key factor in optimising the performance of the graphene-modulated cavity antenna at millimeter-wave frequencies, particularly for applications requiring efficient frequency conversion at higher harmonics.

**Figure 6:**
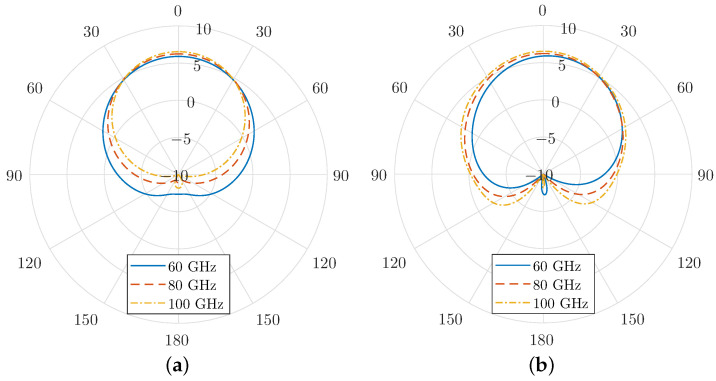
(**a**) E-plane and (**b**) H-plane radiation patterns at different frequencies for a graphene layer harmonic modulation using a constant bias field $E_{0}$ = 300 V/μm and modulation strength $m_{0}$ = 0.9.

Finally, the radiation patterns for both the *E*-plane and *H*-plane are shown in Figure 6 for various harmonic frequencies. These patterns highlight the directional characteristics of the antenna at different harmonic modes. It is evident that the modulation scheme employed in the graphene-coated aperture has minimal impact on the antenna’s core radiation characteristics. The primary lobe, oriented normal to the aperture, remains the dominant feature in the radiation pattern, with a peak gain of approximately 6 dBi. Additionally, the half-power beamwidth (HPBW) is relatively wide, indicating that the antenna maintains strong directional radiation while covering a broad angular range.

Notably, the side-lobe levels remain low across all harmonics, minimising unwanted radiation in off-axis directions. This is a critical performance aspect, as high side-lobes can cause inefficiencies and interference in practical applications. Furthermore, the front-to-back ratio consistently measures around −20 dB, confirming the antenna’s ability to suppress backward radiation effectively. The geometric properties of the SIC antenna support this statement, since the PEC regions cover it entirely except for the open slot on its upper plane. As a result, the forward gain is enhanced, and interference with other components or systems is suppressed.

### 3.2. Periodic Pulse Modulation of Graphene Using Constant Bias

**Figure 7:**
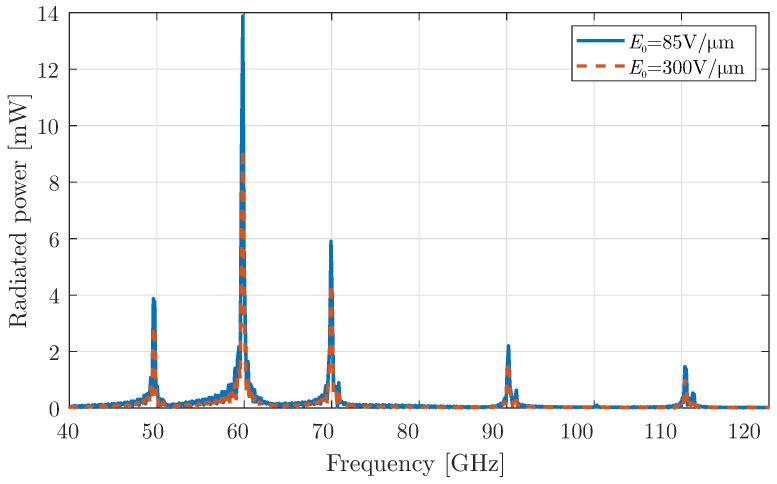
Total radiated power for a graphene layer with periodic pulse modulation using a constant bias field and modulation strength $m_{0}=0.9$.

Continuing our analysis, we now apply the second modulation type of Section 2.1 to graphene, the periodic rectangular pulse. This modulation can be realised using a simple switch, such as am mm-wave PIN diode [37,38], which introduces discontinuities at the “on/off” states and provides a rich spectrum. As in the previous case, the periodic pulse alternates between a constant bias, with the modulation strength again set to $m_{0}=0.9$. The radiated power of the device is depicted in Figure 7. The figure illustrates that the even harmonics are eliminated, and the radiated frequencies are $f_{n}=f_{0}+n\;f_{m}$ GHz, where n=2k+1,k∈Z. An additional interesting feature is the enhancement of the radiated power at higher harmonics, such as at 100 GHz, compared to the simple harmonic modulation of the previous case. An explanation for this behaviour can be attributed to the multiple reflections inside the cavity. In particular, the reflection-transmission system of a PEC wall and a graphene interface acts as a time-varying Fabry–Perot cavity and, under certain dimensions, could enhance the generation of higher-order harmonics.

### 3.3. Harmonic Modulation of Graphene Without Constant Bias

**Figure 8:**
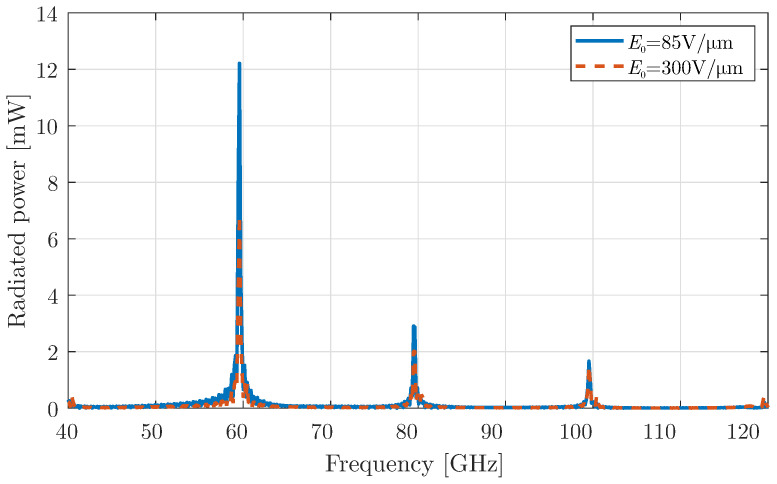
Total radiated power for a graphene layer with harmonic modulation without a constant bias field.

As a final investigation, the harmonic modulation is retrieved, but without a constant bias: specifically, the third modulation type of Section 2.1. Here, the chemical potential values alternate around “zero”, a value that is related to the minimum surface conductivity of graphene. Note that two different values for $E_{1}$, i.e., the peak bias field, are investigated. The extracted results for the radiated power are illustrated in Figure 8, showing that the odd harmonics are now eliminated, and antenna radiates at the frequencies $f_{n}=f_{0}+nf_{m}$ GHz, where n=2k,k∈Z.

This behaviour can be attributed to the even symmetry of the conductivity function with respect to the chemical potential $\mu_{c}$, originating from the frequency-independent term $A_{\mu_{c}}$, i.e., the red solid line shown in Figure 1. In particular, considering an alteration of $\mu_{c}$ around “zero”, $A_{\mu_{c}}$ acquires absolute-like values, which have even symmetry. On the other hand, if the constant bias of Section 3.1 is applied, $\mu_{c}$ obtains only positive values and the dependence on $A_{\mu_{c}}$ is almost linear.

**Figure 9:**
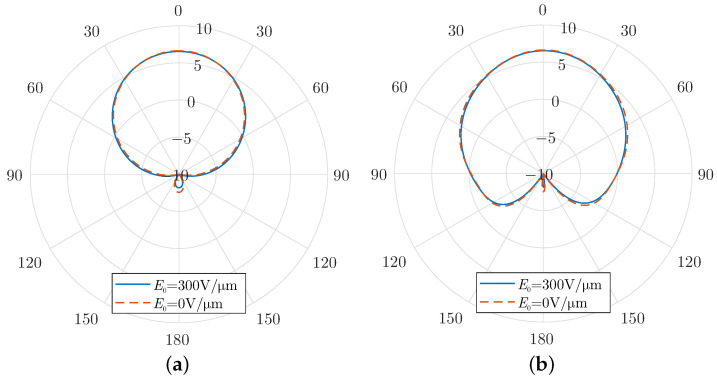
(**a**) E-plane and (**b**) H-plane radiation patterns at 100 GHz for a time-varying graphene layer with and without a constant bias field.

As a consequence, in the absence of constant bias, the surface conductivity is also an absolute-like function with even symmetry. If it is expanded into polynomial terms, only the even terms remain, which are subsequently inherited by the harmonic modulation. As a result, only the even harmonics persist and are considerably enhanced compared to the harmonic modulation case with a constant bias. In particular, the radiated power at 100 GHz is now almost 10 times higher. Finally, Figure 9 compares the radiation patterns for the cases with and without a constant bias. It is evident that the constant bias does not influence the radiation properties significantly, as these depend mainly on the geometric details rather than the bias characteristics.

## 4. Discussion

The time-varying systems have recently received much attention due to their remarkable features, such as harmonic frequency generation. In this context, we proposed an SIC antenna with integrated time-varying graphene on its aperture. This approach operates at the mm-wave/THz spectra, where there is a shortage of sources and other communication components. As a result, it deviates from the related literature which focuses on the microwave spectrum using components with spatio-temporal variations for both frequency generation and anomalous reflection [39,40,41].

Indeed, there are a few papers of time-varying graphene-based devices in mm-wave/THz regime, which can generate harmonics. In [42], the effect on surface wave propagation is studied rather than the radiation in the free space. The latter is examined in [26,43] for periodic graphene arrangements, where the generated harmonics are vanishing after the third order. However, in our work, we propose additionally a mechanism for enhancing these generated harmonics, via the cavity mechanism and the utilisation of additional modulation schemes. As a consequence, harmonics of the fourth and fifth order have a considerable portion of power, as depicted in Figure 7 and Figure 8, respectively.

Finally, a couple of key challenges were identified that can optimise the enhanced frequency conversion. Firstly, a proper geometric arrangement is able to exploit the multiple reflections inside the cavity. This can help to augment certain frequencies, similarly to a Fabry–Perot resonator. Moreover, the application of more advanced modulation schemes along with the inherent time-varying ability of graphene’s chemical potential can amplify the desired harmonics.

## 5. Conclusions

In this paper, we have presented a non-linear antenna design by the integration of a well-documented SIC slot antenna with time-varying graphene, for the generation of higher-order frequencies in the mm-wave spectrum. First, graphene was modelled as having a surface conductivity with Drude dispersion. Time modulation was theoretically introduced in graphene via the alteration of the electric bias field, while time-varying graphene was numerically simulated using an efficient modified FDTD algorithm. Then, the proposed structure was numerically simulated for three different types of time modulation, and results show a successful harmonic generation. Specifically, the higher-order harmonics can be enhanced with a modulation strength larger than 0.7, while the even or odd orders of harmonics can be enhanced by using certain types of periodic modulation of the bias field. Finally, the radiation characteristics of the SIC graphene antenna, designed for the central frequency $f_{0}$, are retained for the neighbouring higher-order harmonics, signifying the effective use of the proposed device for a frequency up-conversion.

As for future work, we aim to equivalently expand the proposed design methodology to include other time-varying surfaces, like space–time metasurfaces. Indeed, 2D arrays of subwavelength particles can be expressed with a surface susceptibility that follows the Lorentz dispersion model; thus, one can utilise the proposed design to include non-linear antennas with time-varying metasurfaces. This type of antenna, if time-modulated elements could be implemented, could be easier to fabricate and ultimately measure for performance. Moreover, additional features could be added on the proposed design for the purpose of filtering or enhancing specific higher-order harmonics. Towards this goal, analytic formulations of the proposed designs as well as extensive use of optimisation or inverse-design techniques would be interesting and beneficial.

## Data Availability

Data available from the authors upon reasonable request.
